# Epithelial–Mesenchymal Transition in Osteosarcoma as a Key Driver of Pulmonary Metastasis

**DOI:** 10.3390/cancers17172922

**Published:** 2025-09-06

**Authors:** Fangcheng Luo, Kosei Ando, Yoshinori Takemura, Tae-Hwi Park, Takafumi Yayama, Shinji Imai

**Affiliations:** Department of Orthopedic Surgery, Shiga University of Medical Science, Otsu 520-2192, Shiga, Japan; luo97@belle.shiga-med.ac.jp (F.L.); takemura@belle.shiga-med.ac.jp (Y.T.); pt100204@belle.shiga-med.ac.jp (T.-H.P.); yayama@belle.shiga-med.ac.jp (T.Y.); simai@belle.shiga-med.ac.jp (S.I.)

**Keywords:** osteosarcoma, EMT, metastasis

## Abstract

Background: Osteosarcoma is an aggressive bone tumor with a high risk of lung metastasis, which severely affects patient survival. EMT plays a major role in tumor spread, therapy resistance, and cancer stemness. This review explores how EMT contributes to osteosarcoma metastasis and the underlying molecular mechanisms. Methods: We reviewed recent studies on EMT-related signaling pathways, transcription factors, and regulatory RNAs in osteosarcoma. We also examined the role of the tumor microenvironment. Results: EMT promotes cell detachment, migration, and lung colonization. Key pathways such as TGF-β, MAPK, PI3K/Akt, STAT3, Notch, and Wnt/β-catenin are involved. Non-coding RNAs further regulate EMT by interacting with these pathways. The tumor microenvironment, including hypoxia and immune cells, also supports EMT and metastasis. Conclusions: EMT is a key driver of metastasis and poor outcomes in osteosarcoma. Targeting EMT and its regulators may help prevent lung spread and improve treatment. Future strategies combining EMT inhibition with existing therapies could be promising for clinical application.

## Simple Summary

Osteosarcoma is a highly aggressive bone tumor that often spreads to the lungs, leading to poor prognosis. This review mainly introduces a biological process called epithelial–mesenchymal transition (EMT), which promotes lung metastasis in osteosarcoma. We describe the related molecules, signaling pathways, and the regulatory role of non-coding RNAs, as well as how the tumor microenvironment affects this process. We also discuss potential therapeutic strategies targeting EMT. A deeper understanding of this process may help suppress metastasis and improve survival in patients with osteosarcoma. Overall, this review shows that EMT plays an important role in lung metastasis of osteosarcoma, and targeting this process may help improve treatment and patient survival.

## 1. Introduction

Osteosarcoma (OS) is a highly aggressive bone malignancy with a bimodal age distribution, peaking in adolescence and after 65 years [1]. It is the most common primary bone tumor in children, with an incidence of 5.6 cases per million in those under the age of 15 years. OS typically develops in the metaphysis of long bones, most frequently in the distal femur, proximal tibia, and proximal humerus [1]. Despite therapeutic advances, the prognosis remains poor for patients with metastatic OS. Thus, new therapies are under investigation. Although the survival rate for localized OS exceeds 60%, the prognosis for metastatic cases remains poor; the survival rates in these cases is below 30%. The lung is the most frequent site of metastasis and consequently determines patient outcomes [2]. Epithelial–mesenchymal transition (EMT) is a key biological process involved in cancer development, tissue repair, and cancer progression. It is regulated by transcription factors, cytokines, and growth factors that drive tumor cell plasticity, invasion, and therapy resistance. EMT also contributes to the formation of cancer stem cells (CSCs) and circulating tumor cells (CTCs) that play critical roles in metastasis and drug resistance [3]. 

The primary treatment for OS is surgical resection, often combined with neoadjuvant and adjuvant chemotherapy for high-grade tumors [4]. Although advancements in surgery and chemotherapy initially improved clinical outcomes, overall survival rates have remained unchanged for decades [5]. The rise of personalized medicine has led to an increasing interest in the development of targeted therapies. In particular, the key pathways, proteins, or molecules for cancer progression have been identified. One critical feature linked to aggressive cancers is EMT. Therefore, in this study, we aim to explore the molecular mechanisms underlying EMT-driven metastasis in osteosarcoma and identify potential therapeutic targets for intervention.

## 2. EMT and Its Role in Cancer Progression

**Figure 1:**
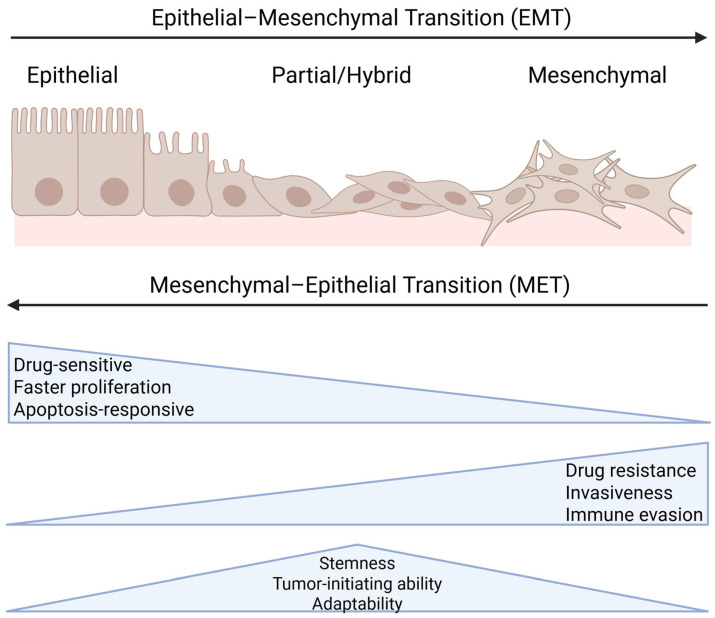
The EMT and MET spectrum. EMT is a dynamic and reversible process in which epithelial cells lose their polarity and cell–cell adhesion, thereby acquiring mesenchymal traits that enhance migration and invasion. This transition occurs along a continuum, with cells often existing in an intermediate partial/hybrid EMT state where they retain both epithelial and mesenchymal characteristics. The reverse process, mesenchymal–epithelial transition (MET), allows mesenchymal-like cells to regain epithelial properties, consequently facilitating tumor colonization at secondary sites. Created with BioRender.com (accessed on 5 September 2025, publication license). Available from: https://BioRender.com/rh1rpfk.

**Figure 2:**
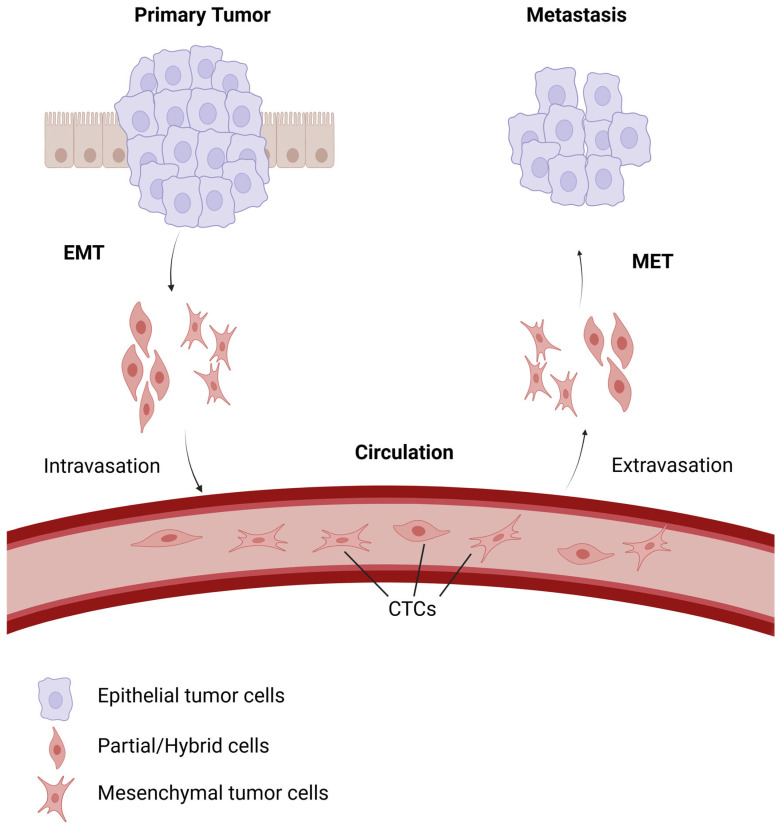
Epithelial–mesenchymal plasticity in metastasis. EMT enables cells from the primary tumor to undergo intravasation and enter the bloodstream as CTCs. These CTCs may exist in epithelial, mesenchymal, or partial/hybrid EMT states. These cells can survive, evade immune detection, and migrate to distant organs while in circulation. MET facilitates extravasation and colonization upon arrival, consequently allowing metastatic tumor formation. Created with BioRender.com (accessed on 5 September 2025, publication license). Available from: https://BioRender.com/6p4p80t.

EMT is a process during which epithelial cells acquire the mesenchymal stem cell phenotype, which facilitates migration, invasion, and metastasis [6] (Figure 1). This process reduces cell–cell adhesion through decreased E-cadherin expression while enhancing cell migration and invasiveness. This allows tumor cells to detach from the primary tumor and enter circulation [7]. CTCs, tumor cells that have entered the bloodstream, often exhibit EMT-like characteristics that enhance their survival in the hostile circulatory environment (Figure 2). EMT enables CTCs to resist apoptosis, evade immune surveillance, activate coagulation pathways, and interact with host cells to promote metastasis [8]. Mesenchymal–epithelial transition (MET) is the reverse process of EMT, in which cells regain epithelial characteristics, including cell polarity, adhesion, and E-cadherin expression (Figure 1). EMT confers increased drug resistance and immune evasion, whereas MET facilitates colonization in distant organs; these processes collectively drive cancer metastasis [9]. 

Partial or hybrid EMT cells represent an intermediate state between epithelial and mesenchymal phenotypes. This allows tumor cells to retain epithelial features while acquiring migratory and invasive abilities [10]. Unlike the traditional view of EMT as a simple switch between epithelial and mesenchymal states, recent studies show that most carcinoma cells undergo incomplete EMT, consequently retaining both epithelial and mesenchymal traits. Epithelial-like cells are sensitive to drugs, grow more rapidly, and respond well to apoptosis signals, whereas mesenchymal-like cells exhibit better drug resistance, invasion, and immune evasion. However, partial or hybrid EMT cells have the highest stemness, tumor-initiation ability, and adaptability, making them key players in metastasis [11]. In addition, both extreme epithelial and fully mesenchymal states may lead to a loss of tumor-initiating and colonization abilities [12]. 

EMT plays a crucial role in cancer progression, particularly in tumor invasion, metastasis, and therapy resistance. Brabletz et al. [13] proposed the migrating CSCs concept and MET hypothesis in 2005: although EMT and CSCs represent different aspects of cancer, they are interconnected processes driving tumor progression and metastatic evolution. In 2008, Mani et al. [14] identified EMT as a key regulator of CSC properties that enhances tumor cell plasticity and survival. Contrastingly, miR-200 family members inhibit EMT; this highlights the role of microRNAs (miRNAs) in EMT regulation [15,16]. In addition, Yu et al. [17] reported that CTCs exhibit dynamic shifts between the epithelial and mesenchymal phenotypes in 2013, suggesting that EMT is not a fixed state but a reversible and plastic process. Furthermore, Zheng et al. [18] demonstrated that EMT is not always required for metastasis but substantially contributes to drug resistance, consequently confirming that EMT is a multifaceted mechanism in cancer progression. These findings underscore the critical role of EMT in tumor dissemination, survival, and therapeutic response, making it a crucial target for future cancer therapies. 

## 3. EMT-Related Signaling Pathways in Cancer

**Figure 3:**
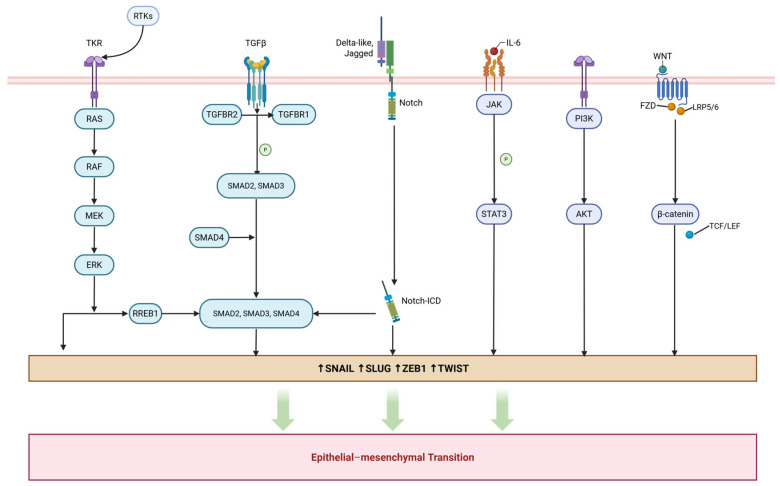
Key signaling pathways involved in epithelial–mesenchymal transition. This diagram illustrates multiple signaling pathways that regulate the expression of EMT transcription factors (SNAIL, SLUG, ZEB1, and TWIST), including RTK, TGF-β, Notch, IL-6/JAK/STAT3, PI3K/AKT, and WNT/β-catenin signaling. These pathways contribute to EMT, a critical process for cancer progression and metastasis. Created with BioRender.com (accessed on 5 September 2025, publication license). Available from: https://BioRender.com/5ieelui.

Most EMT-related pathways converge on common transcription factors (e.g., SNAIL, SLUG, ZEB1, and TWIST) and lead to epithelial marker loss and mesenchymal features; below we summarize pathway-specific mechanisms and therapeutic implications in a concise manner (Figure 3).

### 3.1. TGF-β Signaling Pathway

Transforming growth factor-beta (TGF-β) ligands bind type II receptor (TGFBR2) and recruit/activate the type I receptor (TGFBR1) to initiate downstream cascades [19]. TGFBR1 subsequently phosphorylates receptor-regulated Smads (R-Smads), specifically Smad2 and Smad3. This enables them to form a complex with Smad4 (co-Smad) and translocate into the nucleus, where they regulate the transcription of EMT-related genes [9,19,20,21,22]. There, these complexes bind promoters (e.g., Snail, ZEB1, and Slug) and recruit co-activators such as CBP/p300 to induce EMT programs, together with cytoskeletal/adhesion and polarity changes that favor invasion [20,23]. Functionally, TGF-β suppresses proliferation in early disease but promotes EMT and metastasis in advanced cancers; Smad3 is particularly dominant and controls Snail, ZEB1, Slug, Twist, and FOXC2, which are linked to stemness, drug resistance, and immune evasion [24].

Crosstalk with pathways such as PI3K/Akt, MAPK, and Wnt/β-catenin further enhances the EMT process, underscoring the therapeutic potential of targeting TGF-β signaling [25,26,27]. 

### 3.2. MAPK Signaling Pathway

Growth factors and cytokines trigger RTKs and activate the RAS-RAF-MEK-ERK cascade, in which nuclear ERK influences EMT through transcription factors [28]. One important regulator is RREB1, as MAPK-driven RREB1 helps TGF-β-activated Smad2/3/4 bind to EMT gene promoters, such as SNAIL and fibrogenic genes, thereby strengthening EMT and altering chromatin in a context-dependent way [29,30,31]. Blocking ERK activity prevents RREB1 from recruiting Smads, lowers EMT gene expression, and reduces cell motility, showing that RREB1 provides a functional link between MAPK and TGF-β signaling [29].

### 3.3. Notch Signaling Pathway

When Notch ligands such as Jagged or Delta-like bind to the receptor, the intracellular domain (ICN) is released and moves into the nucleus, where it binds CSL/RBP-Jκ and activates EMT-related transcription [32]. ICN also works together with SMAD proteins, and blocking Notch signaling prevents TGF-β from inducing EMT, which points to Notch as a potential therapeutic target against tumor progression, stemness, and resistance [33].

### 3.4. STAT3 Signaling Pathway

Cytokines activate JAK kinases, which phosphorylate STAT3 and allow its dimerization and nuclear entry to regulate EMT genes [34]. STAT3 drives EMT by inducing Snail, Slug, ZEB1, and Twist, leading to epithelial loss, mesenchymal traits, and increased invasion [35]. It also stabilizes Snail through the LIV-1/GSK3β axis and is maintained by an IL-6/JAK/STAT3 autocrine loop linked to therapy resistance [29,36]. In addition, STAT3 cooperates with TGF-β/SMAD3/4 and activates NF-κB signals that sustain EMT [37].

### 3.5. PI3/Akt Signaling Pathway

The phosphatidylinositol-3-kinase (PI3K)/Akt signaling pathway promotes EMT and malignant behavior by increasing EMT transcription factors and mesenchymal programs [38]. After RTK activation, PI3K converts PIP2 to PIP3, which recruits and activates Akt at Thr308 with the help of PDK1; PTEN reverses this by dephosphorylating PIP3 and limits EMT [39,40]. Akt regulates proliferation, survival, and motility, enhances Twist1 phosphorylation and anti-apoptosis, while its inhibition induces MET, showing Akt as a central EMT regulator [9,41].

### 3.6. Wnt/β-Catenin Signaling Pathway

The Wnt/β-catenin signaling pathway plays a crucial role in EMT, embryogenesis, and cancer progression. In the absence of Wnt ligands, β-catenin is degraded by the AXIN/APC/CK1/GSK-3β complex; ligand binding to FZD/LRP5/6 allows β-catenin to accumulate and enter the nucleus [42,43]. Nuclear β-catenin with TCF/LEF activates EMT drivers such as Snail, Slug, ZEB1, and Twist, while repressing epithelial identity [44]. High β-catenin levels associate with poor prognosis and resistance, and its crosstalk with PI3K/Akt, TGF-β, and Notch further strengthens EMT [9,45]. Inhibitors of β-catenin (ICG-001, PRI-724) and porcupine (WNT974) have shown promise in blocking EMT-related progression [46]. 

## 4. EMT in OS

Osteosarcoma is a tumor of mesenchymal origin, and unlike carcinomas, it does not exhibit the classical epithelial-to-mesenchymal transition characterized by distinct morphological alterations from an epithelial to a mesenchymal phenotype. Reported EMT-like changes in OS cells have been defined mainly by molecular indicators, including reduced expression of E-cadherin, increased expression of mesenchymal markers such as N-cadherin and vimentin, and elevated levels of EMT-associated transcription factors such as Snail, ZEB1, and Twist. Thus, EMT in OS should not be regarded as a phenomenon accompanied by dramatic morphological conversion, but rather as one that is primarily defined by alterations in molecular markers and changes in cellular properties such as invasiveness, motility, and therapy resistance. 

OS is a highly aggressive primary bone malignancy characterized by early pulmonary metastasis, which remains the leading cause of mortality. Approximately 15–20% of patients present with metastases at diagnosis. Of these metastatic lesions, >80% are localized to the lungs [2].The aggressive behavior of OS cells is closely linked to EMT, a dynamic process that enables tumor cells to acquire migratory and invasive properties. OS cells undergo EMT-like transformations despite their mesenchymal origin. This is characterized by upregulated expression of core EMT transcription factors, such as Snail, ZEB1, and Twist. These transcription factors suppress the expression of epithelial markers such as E-cadherin and enhance mesenchymal traits, including N-cadherin and vimentin expression [9]. This phenotypic plasticity enables local tissue infiltration and systemic dissemination, highlighting the critical role of EMT in the progression of OS. 

**Figure 4:**
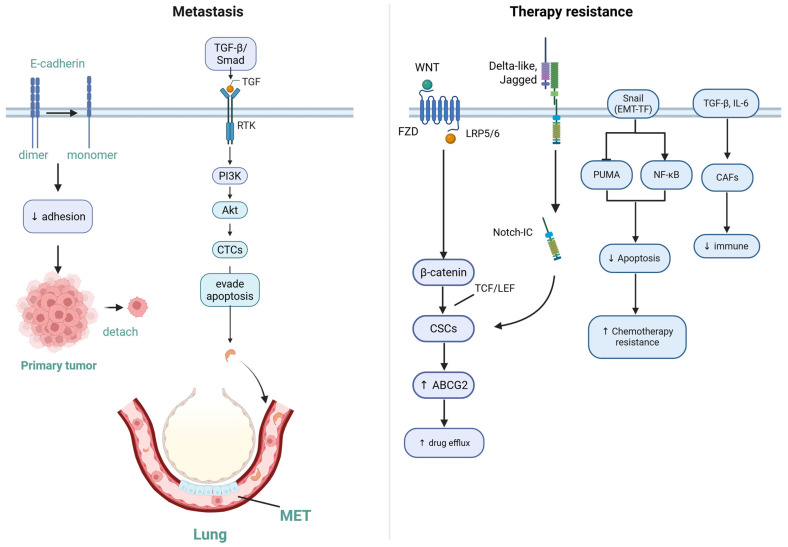
EMT-mediated mechanisms of metastasis and therapy resistance in OS. In osteosarcoma, which is of mesenchymal origin, EMT is defined by molecular and functional changes rather than classical epithelial-to-mesenchymal morphological conversion. EMT promotes metastasis through loss of E-cadherin, reduced adhesion, and tumor cell detachment. Circulating tumor cells survive via TGF-β/Smad–PI3K/Akt signaling, while integrin αvβ3 supports lung colonization and partial MET. EMT also drives therapy resistance by enriching cancer stem cells through Wnt/β-catenin, enhancing drug efflux, inhibiting apoptosis, and remodeling the tumor microenvironment via TGF-β and IL-6. Created with BioRender.com (accessed on 5 September 2025, publication license). Available from: https://BioRender.com/z6jw9zy.

EMT orchestrates the metastatic cascade through sequential mechanisms in OS (Figure 4): 

Primary tumor dissociation: EMT downregulates the expression of cell adhesion molecules, such as E-cadherin, consequently weakening intercellular junctions and enabling the detachment of tumor cells from the primary site [11]. CTC Survival: EMT confers resistance to anoikis, a critical adaptation for CTC survival in circulation. Activation of TGF-β/Smad signaling enhances pro-survival pathways (e.g., PI3K/Akt), allowing CTCs to evade apoptosis [12]. Lung Colonization: EMT increases the expression levels of integrins such as αvβ3, thereby facilitating the attachment of CTCs to lung vasculature. Partial MET later restores epithelial traits, resulting in the progression of metastasis [47]. Single-cell RNA sequencing of OS lung metastases reveals enrichment of EMT-related genes (e.g., SNAI1 and TWIST1), highlighting the dynamic plasticity required for successful colonization [1]. 

EMT contributes to therapy resistance through multifactorial mechanisms: CSC Enrichment: EMT activates Wnt/β-catenin and Notch pathways to induce CSC phenotypes. These cells overexpress drug efflux pumps (e.g., ABCG2), consequently reducing intracellular concentrations of chemotherapeutics (e.g., methotrexate and cisplatin) [3]. Anti-Apoptotic Signaling: EMT transcription factors such as Snail inhibit pro-apoptotic proteins (e.g., PUMA) while activating NF-κB, thereby blunting chemotherapy-induced apoptosis [48]. Microenvironment Remodeling: EMT-driven secretion of TGF-β and IL-6 leads to the recruitment of cancer-associated fibroblasts (CAFs) and immunosuppressive cells. This creates a protective niche that shields tumor cells from therapy [49]. Clinically, high EMT activity correlates with a poor response to standard MAP regimens (methotrexate, doxorubicin, and cisplatin), particularly in pulmonary metastases [50].

## 5. Regulation of EMT in OS: Key Signaling Pathways

### 5.1. TGF-β Signaling Pathway

The TGF-β signaling pathway is a critical regulator of EMT in OS. It facilitates tumor invasion, metastasis, and therapy resistance. TGF-β is widely recognized for its dual role in cancer, acting as a tumor suppressor in early stages and promoting tumor progression in advanced cancers through EMT induction. TGF-β signaling is frequently upregulated in OS, contributing to aggressive tumor behavior and poor prognosis. TGF-β signaling in OS largely follows the canonical Smad-dependent pathway, where ligand binding to TGFBR1/TGFBR2 leads to Smad2/3 phosphorylation, complex formation with Smad4, and nuclear translocation to regulate EMT-associated transcription factors such as Snail, ZEB1, Slug, and Twist in OS cell models [51]. This results in E-cadherin suppression and upregulation of mesenchymal markers such as N-cadherin and vimentin, consequently promoting OS cell plasticity and motility [52]. Additionally, non-Smad pathways, including Wnt/β-catenin and JNK/Smad3, amplify EMT signaling to enhance metastatic potential [53,54]. 

In preclinical studies, given the pivotal role of TGF-β in EMT-mediated OS progression, therapeutic strategies targeting this pathway are under investigation. The inhibition of the TGFβ-induced EMT-associated kinase switch may reverse the chemo-resistance of OSCs to EGFR inhibitors [55]. Oridonin and glaucocalyxin A inhibit EMT and TGF-β1-induced EMT in OS by suppressing the TGF-β1/Smad2/3 signaling pathway [56,57]. Furthermore, activin membrane-bound inhibitor reconstitution inhibited TGF-β-induced EMT; suppressed cell growth, migration, and invasion; and enhanced cisplatin-induced apoptosis in OS cells by downregulating the TGF-β signaling pathway, suggesting its potential as a therapeutic target [58]. 

### 5.2. MAPK Signaling Pathway

The MAPK pathway regulates cell proliferation, survival, and migration in EMT. MAPK signaling is frequently dysregulated in OS, contributing to tumor progression, metastasis, and therapy resistance. Evidence from OS cell models shows that MAPK pathway modulators, including DUSP1, SPRED2, and FGFR1, are involved in EMT regulation in OS, further demonstrating the relevance of this pathway as a therapeutic target [59,60,61]. 

Among the key regulators, Siglec-15 promotes OS progression by activating the DUSP1/MAPK pathway, which enhances EMT and metastatic potential [61]. Similarly, miR-19-mediated downregulation of SPRED2 expression, a negative regulator of MAPK signaling, increases EMT, proliferation, invasion, and migration in OS cells [59]. Additionally, miRNA-133b, which targets FGFR1, has been identified as a tumor suppressor; it inhibits EMT, migration, and invasion while promoting apoptosis [60]. These findings indicate that MAPK signaling acts as a critical driver of EMT-mediated OS aggressiveness. 

Preclinical studies have also evaluated therapeutic strategies. Delphinidin, a natural flavonoid, inhibits EMT through the ERK/p38 MAPK pathway, thereby reducing OS cell motility and invasion [62]. Similarly, lycorine, a plant alkaloid, suppresses OS tumor growth by blocking ERK1/2/MAPK, PI3K/Akt, and Wnt/β-catenin signaling [63]. Furthermore, diosgenin, a steroidal sapogenin, inhibits EMT initiation in OS cells by blocking p38 MAPK, thereby reducing tumor progression [64]. In addition to small molecules, aurora kinase A inhibitor alisertib (MLN8237) exerts pro-apoptotic and pro-autophagic effects in OS by inhibiting p38 MAPK/PI3K/Akt/mTOR signaling, further supporting the therapeutic potential of MAPK inhibition [65]. 

Given its central role in EMT-mediated tumor progression, MAPK inhibition represents a promising therapeutic approach in OS. Small molecule inhibitors, miRNA-based modulation, and natural compounds targeting MAPK components may provide new strategies for the suppression of EMT, reduction in metastasis, and enhancement of sensitivity to chemotherapy.

### 5.3. Notch Signaling Pathway

Notch signaling is another key regulator of EMT. Its activation is linked to tumor progression, metastasis, and chemotherapy resistance in OS. In OS cell models, low-dose chemotherapy, such as low concentrations of doxorubicin and cisplatin, can activate Notch signaling. This promotes EMT and increases tumor cell motility, whereas Notch inhibition reverses these effects [66,67]. 

Beyond chemotherapy-induced EMT, ATG4A overexpression activates Notch, thereby enhancing OS cell migration and invasion [68]. Similarly, SKA3, a spindle-related protein, promotes EMT through Notch activation. Thus, the downregulation of its expression reduces tumor aggressiveness [69]. 

Given its role in EMT and therapy resistance, targeting Notch signaling could be a promising approach for the suppression of metastasis and improvement of chemotherapy response in OS [67,70]. 

### 5.4. STAT3 Signaling Pathway

The STAT3 signaling pathway plays a crucial role in OS progression and EMT regulation. It affects tumor growth, metastasis, and therapy resistance. The JAK2/STAT3 axis has been widely studied in OS, with multiple regulators that either activate or suppress STAT3-mediated EMT identified across OS tissues, cell models, and xenografts [71,72]. Several studies in OS tissues, cell models, and xenografts have highlighted the oncogenic role of STAT3 in EMT regulation. LncRNA NEAT1 promotes OS metastasis and EMT by sponging miR-483, leading to increased STAT3 expression and activation of downstream mesenchymal markers in OS cells [73]. Additionally, tumor-associated macrophages (TAMs) contribute to OS metastasis by activating the COX-2/STAT3 axis, which promotes EMT and enhances invasion potential in cell and mouse models [74]. 

Targeting STAT3 has emerged as a promising therapeutic strategy for the reversal of EMT and suppression of tumor aggressiveness in OS. Pectolinarigenin, a natural compound, inhibits STAT3 signaling via SHP-1, suppressing EMT and metastasis in cell and xenograft models. This leads to the suppression of EMT and tumor metastasis [72]. Piperlongumine, a natural alkaloid, suppresses osteosarcoma cell growth, migration, invasion, and EMT by downregulating the SOCS3/JAK2/STAT3 pathway via inhibition of miR-30d-5p, thereby reducing tumor aggressiveness in OS cells [75]. Furthermore, apatinib, a VEGFR2 inhibitor, suppresses STAT3 activation, consequently reducing OS migration, invasion, and PD-L1 expression. This suggests a role in the prevention of immune evasion [76]. Moreover, irisin inhibits IL-6–induced EMT in OS cells by suppressing STAT3 phosphorylation and Snail expression; this reduces proliferation, migration, and invasion [77]. 

Given the strong link between STAT3 activation and EMT in OS, inhibiting STAT3 signaling represents a viable approach for limiting tumor progression, metastasis, and drug resistance. The use of STAT3 inhibitors, miRNA-based modulation, and natural compounds offers new potential strategies for OS treatment. 

### 5.5. PI3/Akt Signaling Pathway

The PI3K/Akt pathway plays a central role in OS progression, with increasing evidence demonstrating its involvement in EMT, tumor growth, and metastasis. Most of the supporting data comes from OS cell studies. Dysregulation of this pathway, particularly through the loss or downregulation of PTEN expression, has been widely reported in OS, contributing to enhanced malignancy and therapeutic resistance [78]. PI3K/Akt activation promotes EMT by activating key transcription factors, including Snail, Slug, and ZEB1. These transcription factors downregulate E-cadherin expression and upregulate that of mesenchymal markers such as N-cadherin and vimentin, further enhancing OS cell motility and invasion [79,80]. 

Long non-coding RNAs (lncRNAs) and miRNAs are crucial regulators of the PI3K/Akt pathway in OS. The lncRNA RUSC1-AS1 activates PI3K/Akt signaling by targeting miR-340-5p, further promoting tumor migration and invasion [81]. Conversely, tumor-suppressive lncRNAs such as FER1L4 suppress OS progression by inhibiting the PI3K/Akt pathway through miR-18a-5p modulation, leading to reduced EMT and increased apoptosis [82]. Additionally, numerous non-coding RNAs, including miR-802, LncRNA, TDRG1, miRNA-340-5p, LncRNA 691, and MiR-1224-5p, regulate this pathway [83,84,85,86,87,88]. 

In addition to non-coding RNAs, multiple proteins have been implicated in PI3K/Akt-driven EMT in OS. ZCCHC12, an oncogenic factor, enhances PI3K/Akt signaling, consequently driving EMT and increasing OS cell invasion [89]. Some findings have also been confirmed in xenograft models, STEAP2 and fibulin-4 promote OS metastasis via PI3K/Akt/mTOR signaling to reinforce EMT-related changes [90,91]. The tumor-promoting effects of PI3K/Akt are also mediated by key upstream regulators such as IGF1 and CXCL6, which enhance PI3K/Akt signaling to drive OS growth and metastatic progression [92,93]. 

### 5.6. Wnt/β-Catenin Signaling Pathway

Aberrant activation of the Wnt/β-catenin pathway contributes to EMT and OS progression by regulating multiple EMT-associated transcription factors and promoting tumor metastasis. ALOX5AP, a key suppressor of Wnt/β-catenin signaling, inhibits EMT and OS cell invasion. Contrastingly, its low expression is associated with poor prognosis in patient samples [94]. Similarly, microRNA-377-3p suppresses OS progression by targeting CUL1, reducing β-catenin accumulation, and attenuating EMT [95]. In contrast, CCR9 activation facilitates EMT by upregulating the expression of mesenchymal markers such as N-cadherin and vimentin through Wnt/β-catenin signaling, thereby enhancing OS cell migration and invasion in vitro [96]. 

Bone morphogenetic protein 2 has also been implicated in EMT induction through the Wnt/β-catenin pathway, as it enhances the expression of Wnt3α and p-GSK-3β to further promote OS metastasis in cell and animal models [97]. Moreover, lncRNA-CASC15 and MINCR drive EMT by increasing β-catenin nuclear translocation, thereby reinforcing tumor cell invasiveness [98,99]. Downregulation of the expression of PRR11, a protein linked to OS aggressiveness, reduces cell proliferation and invasion by inhibiting Wnt/β-catenin activity, suggesting its role in the regulation of EMT plasticity [100]. 

Given the strong involvement of Wnt/β-catenin signaling in OS EMT and metastasis, therapeutic approaches targeting this pathway have been explored. Isoquercitrin, a plant-derived flavonoid, suppresses OS progression by inhibiting β-catenin activation and reducing the expression of EMT markers [101]. Similarly, ginsenoside Rg3 blocks EMT by downregulating Wnt/β-catenin and Snail expression, ultimately impairing OS migration and invasion in cell and xenograft models [102]. Moreover, zinc oxide (ZnO) nanoparticles inhibit OS metastasis by downregulating β-catenin expression via the HIF-1α/BNIP3/LC3B-mediated mitophagy pathway, offering a potential therapeutic strategy against OS in in vitro and in vivo studies [103]. 

## 6. The Interplay Between EMT and the Tumor Microenvironment in OS

The tumor microenvironment is a complex ecosystem comprising stromal cells, immune cells, the extracellular matrix (ECM), and signaling molecules, all of which synergistically drive EMT and metastasis in tumors [104]. Here, we dissect the dynamic crosstalk between EMT and key tumor microenvironment components, highlighting their roles in fostering aggressive tumor behavior and therapeutic resistance. 

CAFs promote EMT and remodel the tumor microenvironment during OS progression. These cells are highly enriched in recurrent OS and regulate EMT through lysyl oxidase, which enhances OS cell invasion and metastasis [105]. Additionally, CAF-targeting therapies, such as sulfatinib, inhibit the differentiation of skeletal stem cells into CAFs and block fibroblast growth factor secretion, thereby suppressing EMT and OS progression [106]. 

TAMs are integral to the establishment of an immunosuppressive tumor microenvironment and promotion of OS metastasis through EMT. They enhance OS aggressiveness by upregulating the lncRNA PURPL/miR-363/PDZD2 axis, which suppresses miR-363 to elevate PDZD2 expression, thereby driving tumor proliferation, invasion, and EMT [107]. Additionally, a HMGB1-RAGE-mediated positive feedback loop between OS cells and TAMs reinforces M2 macrophage polarization, further amplifying EMT and metastatic potential through reciprocal activation of high mobility group box 1 (HMGB1) and receptor for advanced glycation end product (RAGE) signaling [108]. Exosomes derived from OS cells can induce M2 macrophage polarization via the Tim-3 pathway, further enhancing tumor migration, invasion, and EMT [109]. Mechanistically, TAMs promote EMT through the COX-2/STAT3 axis, leading to upregulation of the expression of EMT transcription factors and enhanced metastatic potential [74].

In the hypoxic microenvironment, hypoxia-inducible factor-1α (HIF-1α) drives EMT by activating transcription factors such as Snail and Twist. This consequently facilitates tumor cell invasion and metastasis. Moreover, HIF-1α boosts aerobic glycolysis, known as the Warburg effect, by increasing the expression of glucose transporters such as GLUT1 and that of key glycolytic enzymes such as LDHA and HK2, thereby maintaining tumor cell energy metabolism and survival. HIF-1α forms a positive feedback loop with SENP1, further stabilizing its protein expression and reinforcing EMT [110]. Additionally, HIF-1α downregulates β-catenin expression via BNIP3/LC3B-mediated mitophagy or promotes angiogenesis and metastasis through the mTOR/HIF-1α/VEGF signaling axis [103,111]. Targeted interventions such as resveratrol and tetrahydrocurcumin can reverse the EMT phenotype by inhibiting HIF-1α protein accumulation. In contrast, ZnO nanoparticles and ginsenoside Rg3 suppress metabolic reprogramming and metastasis by disrupting downstream HIF-1α pathways. These findings demonstrate that HIF-1α is a central regulator of hypoxic adaptation and a promising therapeutic target [103,111,112,113]. 

## 7. EMT-Targeting Non-Coding RNAs as Therapeutic Tools

Non-coding RNAs, including miRNAs, lncRNAs, and circular RNAs (circRNAs), have emerged as key regulators of EMT in OS. As they modulate EMT-associated pathways, these non-coding RNAs can serve as potential therapeutic targets for the inhibition of tumor progression or enhancement of sensitivity to treatment. 

The following tables provide an overview of EMT-regulating non-coding RNAs in OS, categorized by their type, regulatory function, targeted pathways, and therapeutic potential. The following tables summarize representative miRNAs [52,55,59,60,83,86,88,95,114,115,116,117,118,119,120,121,122,123,124,125,126,127,128,129,130,131,132,133,134,135,136,137,138,139,140,141,142,143,144,145,146,147,148,149,150,151,152,153,154,155,156,157,158,159] in Table 1, lncRNAs [71,73,80,98,99,157,160,161,162,163,164,165,166,167,168,169,170,171,172,173,174,175,176,177,178,179,180,181,182,183,184,185,186,187,188,189,190,191,192,193,194,195,196,197,198,199,200,201,202,203,204,205,206,207,208,209,210,211,212,213,214,215,216] in Table 2, and circRNAs [217,218,219,220,221,222,223,224,225,226,227] in Table 3; full comprehensive lists are provided in Supplementary Tables S1–S3.

## 8. Conclusions

OS is a highly aggressive primary bone malignancy, with lung metastasis as a key factor in poor prognosis. This review systematically discusses the central role of EMT in OS metastasis and its molecular mechanisms. EMT promotes tumor cell detachment from the primary site, resistance to anoikis, and distant colonization by regulating key transcription factors (e.g., Snail, ZEB1, and Twist) and downstream signaling pathways (e.g., TGF-β, MAPK, and Wnt/β-catenin). Interactions between EMT and the tumor microenvironment (e.g., CAFs, TAMs, and hypoxia) further enhance tumor invasiveness and therapy resistance. Additionally, non-coding RNAs (miRNA, lncRNA, and circRNA) regulate EMT-related pathways and have demonstrated therapeutic potential. For example, miR-429 suppresses ZEB1, lncRNA NEAT1 activates STAT3, and circRNAs modulate signaling networks through competitive miRNA binding. 

Challenges remain despite significant advances in understanding the complex EMT regulatory network in OS. First, the dynamic and heterogeneous nature of EMT may limit the efficacy of single-target therapies. Second, the interplay between EMT and the tumor microenvironment requires further investigation. Finally, the clinical translation of EMT-targeting strategies (e.g., small-molecule inhibitors of TGF-β or STAT3) needs validation for safety and efficacy. Future research should focus on multi-target combination therapies by integrating EMT inhibitors with immunotherapy or chemotherapy. Moreover, single-cell sequencing and spatial transcriptomics can elucidate the spatiotemporal heterogeneity of EMT, guiding precision treatment strategies. Thus, targeting core EMT pathways and their microenvironmental regulators may provide new therapeutic directions, consequently overcoming treatment bottlenecks and improving patient outcomes. 

## Figures and Tables

**Table 1:** Representative miRNAs Targeting EMT in OS.

Non-Coding RNA	Type	Regulatory Function on EMT	Target Genes/Pathways	Therapeutic Potential	Delivery Strategy	References
MiR-19	miRNA	Promotes	SPRED2, ERK/MAPK, Autophagy	Enhances OS proliferation, invasion, migration, and EMT	miRNA inhibitors	PMID: 33023313 [59]
MiR-135b	miRNA	Promotes	TAZ, Hippo pathway, EMT markers	Targeting miR-135b may suppress OS proliferation, EMT, and metastasis	miRNA inhibitors	PMID: 28823959 [133]
MiR-486	miRNA	Suppresses	PIM1, EMT markers	Targeting miR-486 may inhibit OS invasion and EMT	miRNA mimics	PMID: 30103304 [137]
MiR-429	miRNA	Suppresses	ZEB1, EMT markers (E-cadherin, Vimentin, N-Cadherin, Snail)	Targeting miR-429 may inhibit OS progression and metastasis	miRNA mimics	PMID: 28694763 [152]
MiR-128	miRNA	Suppresses	ITGA2, EMT markers	Targeting miR-128 may inhibit OS migration, invasion, and EMT	miRNA mimics	PMID: 26700675 [138]
MiR-22	miRNA	Suppresses	Twist1, EMT markers	Targeting miR-22 may inhibit OS EMT and progression	miRNA mimics	PMID: 32391253 [114]
MiR-155	miRNA	Promotes	TNF-α, ERK signaling, CSC markers (CD24, CD90, CD133)	Targeting miR-155 may inhibit OS CSC transformation and EMT	miRNA inhibitors	PMID: 31669646 [122]

This table includes representative miRNAs regulating EMT in osteosarcoma.

**Table 2:** Representative lncRNAs Targeting EMT in OS.

Non-Coding RNA	Type	Regulatory Function on EMT	Target Genes/Pathways	Therapeutic Potential	Delivery Strategy	References
ZEB2-AS1	lncRNA	Promotes	ZEB2-AS1 pathway	Promotes OS proliferation, EMT, migration, and invasion	Knockdown	PMID: 33085924 [174]
HOTAIR	lncRNA	Suppresses	LPR5, Wnt/β-catenin signaling	Suppresses OS migration, invasion, and proliferation	Overexpression	PMID: 36816362 [202]
MALAT1	lncRNA	Promotes	miR-590-3p	Promotes OS proliferation, migration, invasion, and EMT	Knockdown	PMID: 36277152 [166]
KIAA0087	lncRNA	Suppresses	miR-411-3p/SOCS1/JAK2/STAT3	Inhibits OS growth, metastasis, and EMT	Overexpression	PMID: 37009803 [71]
TUG1	lncRNA	Promotes	miR-144-3p/EZH2/Wnt/β-catenin	Promotes OS tumorigenesis, migration, and EMT	Knockdown	PMID: 28902349 [157]
FER1L4	lncRNA	Suppresses	miR-18a-5p/SOCS5, PI3K/AKT	Induces apoptosis, inhibits EMT and PI3K/AKT activation	Overexpression	PMID: 31473323 [80]
CRNDE	lncRNA	Promotes	Wnt/β-catenin pathway	Enhances OS proliferation, invasion, and EMT	Knockdown	PMID: 31898343 [211]

This table includes representative lncRNAs regulating EMT in osteosarcoma.

**Table 3:** Representative circRNAs Targeting EMT in OS.

Non-Coding RNA	Type	Regulatory Function on EMT	Target Genes/Pathways	Therapeutic Potential	Delivery Strategy	References
Circ_0001721	circRNA	Promotes	miR-372-3p/MAPK7	Enhances OS progression, migration, invasion, and EMT	Knockdown	PMID: 32982424 [224]
Circ-FOXM1	circRNA	Promotes	miR-320a/miR-320b/FOXM1/Wnt	Enhances OS proliferation, migration, invasion, and EMT	Knockdown	PMID: 35799265 [217]
Circ-CDR1as	circRNA	Promotes	miR-7/EGFR/CCNE1/PI3KCD/RAF1	Enhances OS proliferation, migration, invasion, and EMT	Knockdown	PMID: 30425578 [220]
CircMYO10	circRNA	Promotes	miR-370-3p/RUVBL1/Wnt/β-catenin	Enhances OS proliferation, chromatin remodeling, and EMT	Knockdown	PMID: 31665067 [227]
CircMGEA5	circRNA	Promotes	miR-153-3p/miR-8084/ZEB1/Snail	Enhances OS metastasis, EMT, and invasion	Knockdown	PMID: 36564929 [223]
CircPRKAR1B	circRNA	Promotes	miR-361-3p/FZD4/Wnt	Enhances OS proliferation, migration, invasion, and EMT	Knockdown	PMID: 34716310 [225]
Circ_0078767	circRNA	Suppresses	miR-889/KLF9	Inhibits OS proliferation, migration, invasion, and EMT	Overexpression	PMID: 35758280 [226]
CircUBAP2	circRNA	Promotes	miR-641/YAP1	Enhances OS proliferation, invasion, and EMT	Knockdown	PMID: 32528231 [221]
Circ_0021087	circRNA	Suppresses	miR-184/FOSB	Inhibits OS proliferation, migration, invasion, and EMT	Overexpression	PMID: 34076278 [218]

This table includes representative circRNAs regulating EMT in osteosarcoma.

## Data Availability

No new data were created or analyzed in this study. Data sharing is not applicable to this article.

## Supplementary Materials

The following supporting information can be downloaded at https://www.mdpi.com/article/10.3390/cancers17172922/s1, Table S1: miRNAs Targeting EMT in OS; Table S2: lncRNAs Targeting EMT in OS; Table S3: circRNAs Targeting EMT in OS.

## Abbreviations

The following abbreviations are used in this manuscript:

OS	Osteosarcoma
EMT	Epithelial–Mesenchymal Transition
TGF-β	Transforming Growth Factor-beta
RTK	Receptor Tyrosine Kinase
MAPK	Mitogen-Activated Protein Kinase
PI3K	Phosphoinositide 3-Kinase
AKT	Protein Kinase B
mTOR	Mechanistic Target of Rapamycin
miRNA	MicroRNA
lncRNA	Long Non-Coding RNA
circRNA	Circular RNA
ceRNA	Competing Endogenous RNA
TF	Transcription Factor
ncRNA	Non-Coding RNA
